# Safety and Feasibility of US-guided Microwave Ablation for the Treatment of Bethesda III Thyroid Nodules with Negative Eight-Gene Panel Mutational Profile

**DOI:** 10.1148/rycan.240058

**Published:** 2024-12-13

**Authors:** Qingqing Tang, Jiawei Chen, Dengke Zhang, Qingnan Huang, Yong Chen, Xuexin Liang, Kai Zeng, Yuxian Guo, Mingliang Huang, Yanghui Wei

**Affiliations:** From the Department of Surgery, The Eighth Affiliated Hospital, Sun Yat-sen University, 3025 Shennan Middle Road, Futian District, Shenzhen 518033, China.

**Keywords:** Ablation Techniques, Radiation Therapy/Oncology, Head/Neck, Thyroid, Safety, Observer Performance

## Abstract

**Purpose:**

To evaluate the safety and efficacy of US-guided thermal ablation in the treatment of Bethesda III thyroid nodules with negative eight-gene panel testing results.

**Materials and Methods:**

This retrospective single-center study included patients with thyroid nodules diagnosed as Bethesda category III (atypia of undetermined significance) at fine-needle aspiration biopsy and with negative eight-gene testing results who were treated with US-guided microwave ablation (MWA) between July 2020 and September 2023. Incidence of complications, technical success rate (TSR), volume reduction rate (VRR), nodule recurrence, and thyroid function were evaluated over a follow-up period of 2 years. Data before and after MWA were compared using variance analysis and the Cochran-Mantel-Haenszel χ^2^ test.

**Results:**

A total of 101 Bethesda III nodules were detected in 95 patients (mean ± SD age, 47.08 years ±14.63; 79 female patients, 16 male patients), all of which were completely ablated (100% TSR). Two patients experienced mild neck swelling and pressure sensation after the minimally invasive operation, and the incidence of postoperative complications was 2% (two of 95). None of the patients experienced tumor recurrence or progression. At 2-year follow-up, the mean VRR of the ablated area was 90.88% ± 13.59 in 15 patients; 87% (13 of 15) of these patients had a 100% VRR. There was no evidence of a difference in thyroid function before and after MWA from 1 to 24 months (*P* = .15–.99).

**Conclusion:**

US-guided MWA was safe and effective for the treatment of Bethesda III thyroid nodules with negative eight-gene panel testing results.

**Keywords:** Ablation Techniques, Radiation Therapy/Oncology, Head/Neck, Thyroid, Safety, Observer Performance

Published under a CC BY 4.0 license.

See also commentary by Faintuch and Sacks in this issue.

## Graphical abstract

**Figure:**
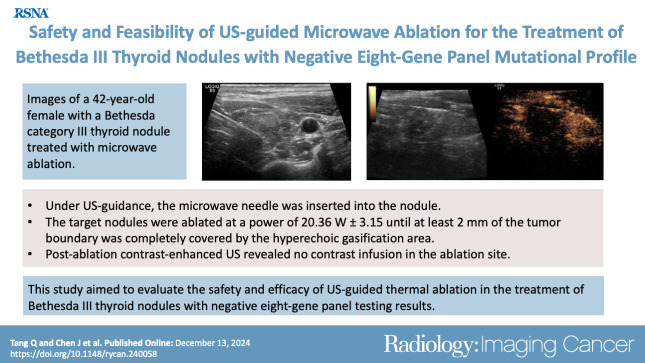

**Summary**

US-guided microwave ablation was safe and effective in treating patients with Bethesda category III thyroid nodules with negative polygenic panel tests and did not increase the risk of tumor progression.

**Key Points**

■ In a retrospective study of 95 patients with Bethesda III thyroid nodules (atypia of undetermined significance) treated with US-guided microwave ablation, the ablation success rate was 100% (95 of 95).
■ None of the patients had tumor recurrence or progression during follow-up, and there was no evidence of a difference in thyroid function before and after ablation (*P* = .15–.99).
■ At the 2-year follow-up, the mean ± SD volume reduction rate (VRR) of the ablated area in 15 patients was 90.88% ± 13.59, with 13 of these patients showing a VRR of 100%.

## Introduction

Thyroid nodules are commonly detected in adults at physical examination and US examination, with prevalences of 5%–7% and up to 65%, respectively (1). US-guided fine-needle aspiration biopsy (FNAB) is an important method for evaluating benign and malignant thyroid nodules, with interpretation of FNAB results using the Bethesda System for Reporting Thyroid Cytopathology. The 2023 Bethesda System for Reporting Thyroid Cytopathology has named the six diagnostic categories of FNAB: (I) nondiagnostic; (II) benign; (III) atypia of undetermined significance; (IV) follicular neoplasm; (V) suspicious for malignancy; and (VI) malignant (2). Bethesda category III nodules are referred to as atypical lesions of undetermined significance and account for 20%–30% of FNAB results. Accurately assessing the malignancy risk of these nodules remains challenging. Previous studies report an estimated malignancy risk of 6%–18% (2); whereas, in China, the risk ranges from 45.5% to 71.4% (3).

Eight-gene molecular testing can help to increase diagnostic accuracy of Bethesda III nodules. Typical molecular markers associated with thyroid cancer include *BRAF* mutations, *RAS* mutations, *RET* or *PTC* rearrangements, *TERT* promoter mutations, *TP53* mutations, and *PAX8/PPAR *γ rearrangements (4). Detection of these mutations is strongly correlated with malignant outcomes. *BRAF V600E* mutation occurs in 35%–77% of papillary thyroid cancers, with a malignancy risk of 98.9%–100% (5–8). Estimated risk of malignancy is 87% for *RAS* mutation-positive Bethesda III nodules and greater than 95% for *RET/PTC* or *PAX8/PPAR *γ mutation-positive Bethesda III nodules (9). Therefore, Bethesda III nodules with positive genetic mutation results are usually considered to be high-risk nodules. Bethesda III nodules with negative genetic mutation results have only a 6% malignancy risk, and the risk of cancer spreading outside the thyroid is less than 1% (9). However, patients who undergo regular follow-up may experience psychologic stress, and there is also a potential risk of cancer progression and metastasis (10). For some patients who choose surgical treatment, the associated surgical risk ranges from 5% to 40% (11–13).

Thyroid thermal ablation therapy is generally considered as a more aggressive but less invasive and risky method to treat Bethesda III nodules with negative genetic mutation results. In 2023, the American Thyroid Association issued a statement that listed thyroid nodules with cytologically indeterminate biopsy result and negative molecular markers as relative contraindications for thermal ablation (14,15). This assessment should require further investigation in clinical trials. To date, only two studies have been published on radiofrequency ablation for the treatment of atypical thyroid nodules (16,17). Further evidence of the efficacy and safety of ablation therapy will bring confidence to patients and clinical decision-makers.

The purpose of this study was to determine the safety and efficacy of US-guided microwave ablation (MWA) for patients with Bethesda III thyroid nodules with negative molecular testing results.

## Materials and Methods

### Study Design and Sample

**Figure 1:**
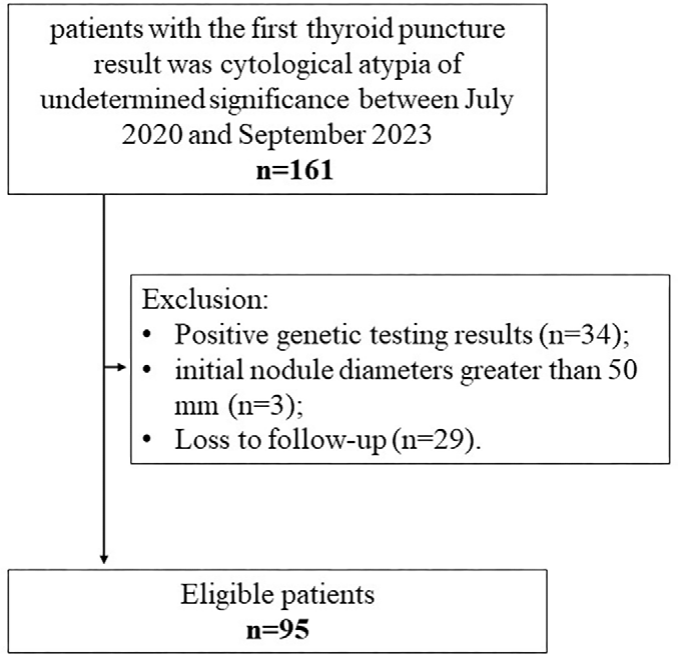
Flowchart shows patient selection.

This retrospective study was approved by the Ethics Review Committee of the Eighth Affiliated Hospital of Sun Yat-sen University (No: 2020–004–03), and the requirement for informed consent was obtained. It was conducted in accordance with local institutional guidelines and regulations, as well as the Declaration of Helsinki for human research. The private information of each patient was protected. Patients with thyroid nodules who were initially diagnosed at the Eighth Affiliated Hospital of Sun Yat-sen University from July 2020 to September 2023 were assessed for eligibility. Patients were included if they: *(a)* had FNAB-confirmed atypia of undetermined significance nodules; *(b)* underwent eight-gene panel testing; *(c)* had US findings of single or multiple lesions, less than 50 mm in diameter; *(d)* underwent MWA therapy; and *(e)* had no family history of thyroid cancer and no history of neck radiation exposure during adolescence or childhood. Exclusion criteria were as follows: *(a)* genetic testing indicating any mutation; *(b)* tumor close to or invading the capsule; *(c)* suspicious metastatic lymph nodes or distant metastasis in the neck; *(d)* previous thyroid dysfunction, including hyperthyroidism and hypothyroidism; *(e)* no follow-up after MWA (Fig 1). All patients were initially referred to the thyroid surgery ward for risk assessment. They were fully informed of the benefits and risks of operation and the potential malignancy risk of Bethesda III nodules. They were also informed that current guidelines did not recommend MWA treatment. Despite this, they still refused surgery and strongly requested MWA.

### Instruments

Thyroid eight-gene panel detection was performed using the fluorescence polymerase chain reaction method (Table 1). Genetic testing was performed for patients with Bethesda III nodules diagnosed by FNAB after full informed consent was received.

**Table 1:**
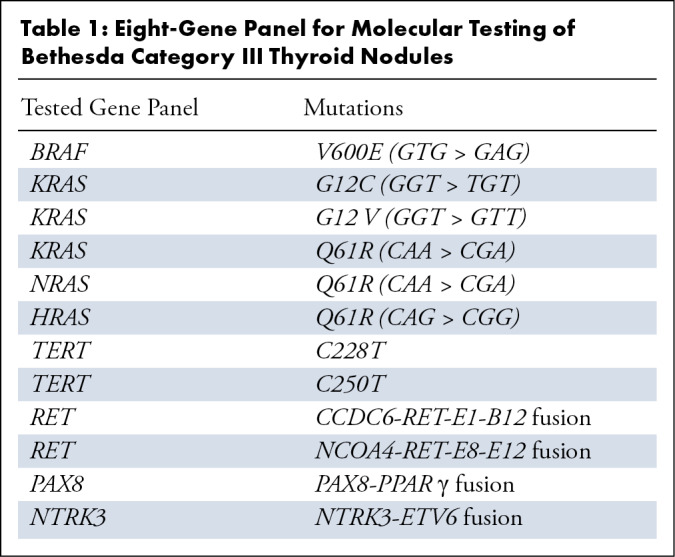
Eight-Gene Panel for Molecular Testing of Bethesda Category III Thyroid Nodules

### MWA Procedure

Three sets of US diagnostic instruments were used: Hitachi Arietta 70, SonoScape P50 Pro, or Philips EPIQ 7–1. The probe center frequency was 7 MHz. Patients underwent contrast-enhanced (sulfur hexafluoride microbubbles [59 mg/vial]) US following MWA to evaluate efficacy of the procedure. The XR-A and XR-B systems (Nanjing Great Wall Information System) were used to perform MWA.

**Figure 2:**
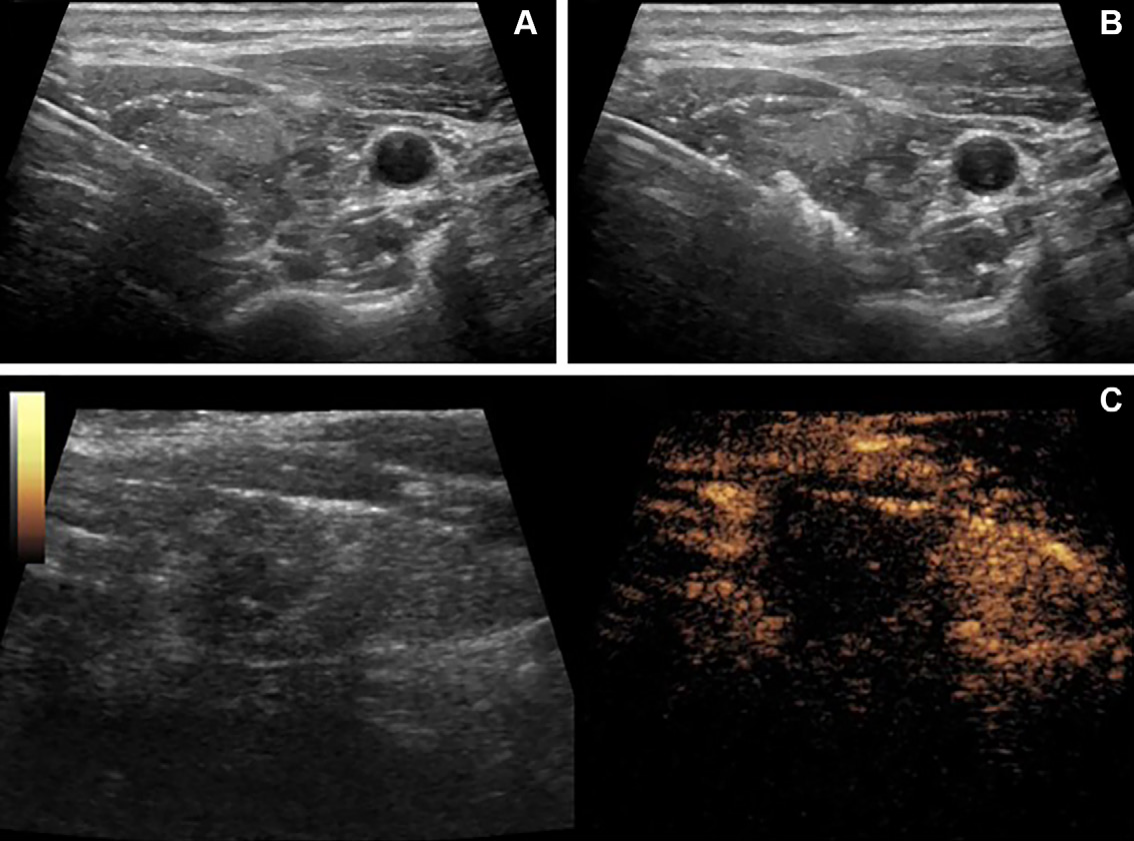
US images in a 42-year-old woman with a Bethesda III thyroid nodule treated with microwave ablation. **(A)** Under US guidance, the microwave needle is inserted into the nodule. **(B)** The target nodules were ablated at a power of 20.36 W ± 3.15 until at least 2 mm of the tumor boundary was completely covered by the hyperechoic gasification area. **(C)** Postablation contrast-enhanced US revealed no contrast material infusion in the ablation site.

The patient was placed in a supine position with full exposure to the anterior cervical region. The thyroid nodules were routinely disinfected and anesthetized after US localization. Before ablation, the operator (Y.W., with >10 years of experience in thyroid surgery and proficiency in US) carefully evaluated the relationship between the nodule and the key structures of the neck and designed the optimal puncture point and needle path. Real-time US was used to monitor the needle trajectory and needle tip position. Under US guidance, the microwave needle was inserted into the nodule with a mean power setting of 20.36 W ± 3.15, and ablation was performed in 8-second durations. The ablation frequency depended on nodule size. For smaller nodules, fixed ablation was used, whereas for larger nodules, a moving-shot ablation technique was used to cover the area except the 2 mm to the capsule boundary (Fig 2). Immediately after ablation, contrast-enhanced US was used to evaluate the efficacy of MWA. Complete ablation was defined as the absence of contrast medium perfusion in the ablation lesion. Areas of nodular enhancement in the ablated lesion were considered as tumor residue and additional treatment was performed. After the procedure, local pressure was applied to the puncture site and needle trajectory for 30 minutes, and the patient was sent back to the ward for 24-hour monitoring of vital signs for any abnormalities. After confirming the absence of complications, such as bleeding and hoarseness, the patient was discharged.

### Observation and Follow-up of Relevant Indicators

Each patient’s physical signs during the procedure, the time and energy used for MWA, and the nodule volume before and after ablation were recorded. The occurrence and duration of complications (eg, skin burns, neck swelling, pain, voice changes, limb numbness) was also recorded. Follow-up was conducted every 3 months in the 1st year after MWA. Thyroid function and US examination were reviewed every 6 months in the 2nd year after the procedure to screen for recurrence and suspected lesions, as well as cervical lymph node metastasis. The volume and shrinkage rate of the ablation area were recorded during each follow-up visit using the volume calculation formula (V = πabc/6, where V is the volume, and a, b, and c are the three diameters of the nodule) and volume reduction rate (VRR) formula (VRR = ([initial volume − final volume])/initial volume × 100%). If the ablation area only left linear scars and the volume could not be calculated, it was considered to be completely absorbed. The effectiveness of thermal ablation was defined as the absence of abnormal enhancement at contrast-enhanced US within 6 months. If suspicious lesions were found, FNAB was performed for definitive diagnosis. The cosmetic score was determined by a clinician (Y.W.) with more than 10 years of experience in thyroid surgery as the following: no palpable mass (score 0); no cosmetic problem but palpable mass (score 2); easily visible mass (score 3).

### Statistical Analysis

General clinical data before MWA, operation time, complications, follow-up data after MWA, and thyroid function after MWA were collected and collated using Microsoft Excel 2021. SPSS version 25 and GraphPad Prism version 9.3.0 were used for statistical analysis and charting. Data measurements are expressed as mean, range, and SD. Variance analysis and Cochran-Mantel-Haenszel χ^2^ test were used to compare data before and after ablation. *P* < .05 was considered statistically significant.

## Results

### Patient Characteristics

A total of 161 patients with Bethesda III nodules were initially considered for inclusion in the study. Patients were excluded for positive molecular testing results (*n* = 34), initial nodule diameters greater than 50 mm (*n* = 3), and a lack of follow-up after MWA (*n* = 29). Thus, 95 patients with 101 Bethesda III nodules and negative molecular testing were included in the final study sample (Fig 1). Patients had a mean age of 47.08 years (range, 11–78 years; SD, 14.63), with 79 female patients (83%, 79 of 95) and 16 male patients (17%, 16 of 95). The average nodule volume was 2947.05 mm^3^ (range, 6.30–23440.73 mm^3^; SD, 4696.83), and the average maximum diameter of the nodules was 17.24 mm (range, 2.90–44 mm; SD, 10.99). The baseline characteristics of the lack of follow-up patients (*n* = 29) and eligible patients (*n* = 95), including age, sex, and maximum diameter and the volume of the nodules were similar between the two groups (all *P* > .05). This finding indicated that excluding these patients from our cohort has no significant impact on our result. Table 2 shows the clinical characteristics of eligible patients and the lack of follow-up patients.

**Table 2:**
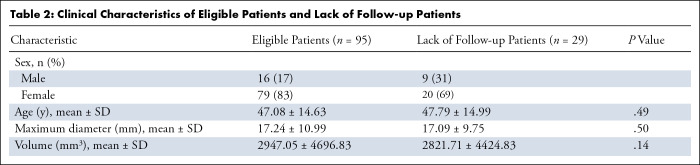
Clinical Characteristics of Eligible Patients and Lack of Follow-up Patients

### Ablation Outcome

**Figure 3:**
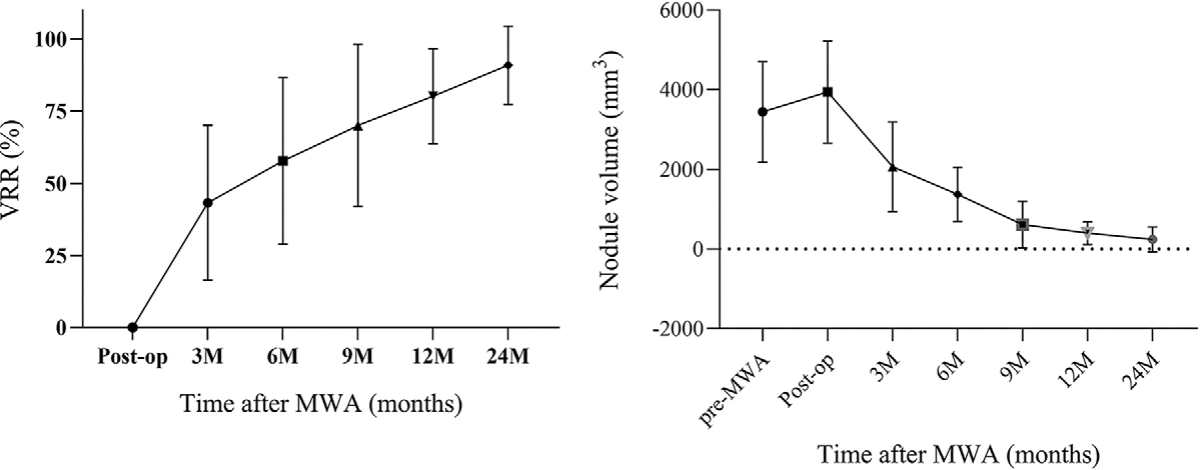
Graphs show the variations of volume reduction rate (VRR) and nodule volume before and after microwave ablation (MWA). Error bars in graphs represent SDs. The different shapes of points represent mean values of VRR at different times after MWA. The dotted line in the nodule volume graph indicates Y = 0.

Table 3 shows lesion characteristics before and after MWA. All lesions were completely ablated successfully in one session, for a technical success rate of 100%. The average follow-up time of the 95 patients was 6.83 months, with the longest follow-up time of 24 months. The efficacy of MWA with respect to volume reduction was assessed. Final VRR within 2-year follow-up is shown in Figure 3. The volume of the nodules in the ablation area was reduced compared with preablation volume. Among the 36 patients who completed the 1-year follow up, mean VRR was 83.54% ± 15.53, with 100% VRR observed in 56% (20 of 36) of patients. After the 2-year follow-up, 15 patients had a mean VRR of 90.88% ± 13.59, with 100% VRR observed in 87% (13 of 15) of patients. Nodule volume reductions were accompanied by cosmetic improvements. There was a linear-by-linear association between cosmetic score and follow-up time (χ^2^ = 69.23, *P* < .001, *R* = −0.42), which suggests a moderate negative correlation between cosmetic score and follow-up time, indicating that patients’ neck appearance improved with longer follow-up time.

**Table 3:**
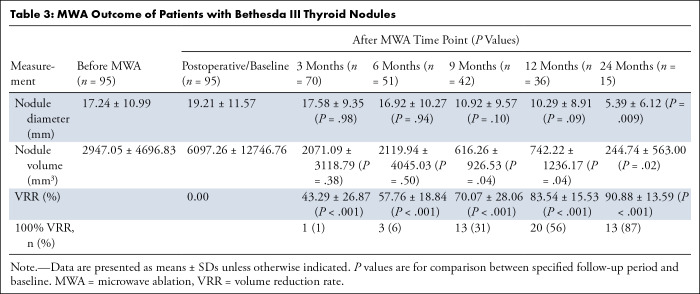
MWA Outcome of Patients with Bethesda III Thyroid Nodules

**Figure 4:**
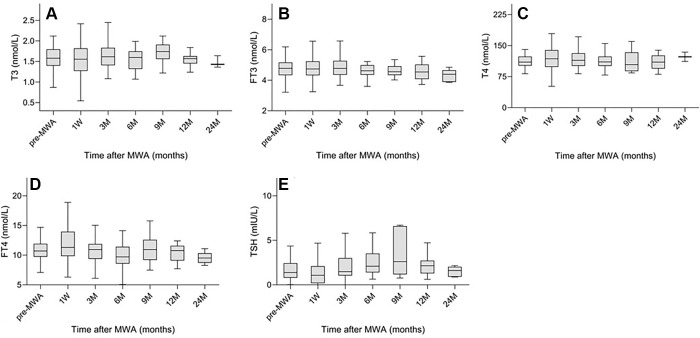
Box and whisker plots of the variations of **(A)** triiodothyronine (T3), **(B)** free triiodothyronine (FT3), **(C)** thyroxine (T4), **(D)** free thyroxine (FT4), and **(E)** thyroid-stimulating hormone (TSH) before and after microwave ablation (MWA). Collectively, there was no evidence of differences in T3, FT3, T4, FT4, and TSH levels at the different follow-up periods compared with before the MWA. The ends of the box are the upper and lower quartiles; a vertical line inside the box marks the median; the two lines outside the box are the whiskers extending to the highest and lowest observations.

Thyroid function analysis data before and after ablation are shown in Table 4. Collectively, levels of triiodothyronine, free triiodothyronine, thyroxine, free thyroxine, and thyroid-stimulating hormone did not change significantly over the follow-up period (Fig 4).

**Table 4:**
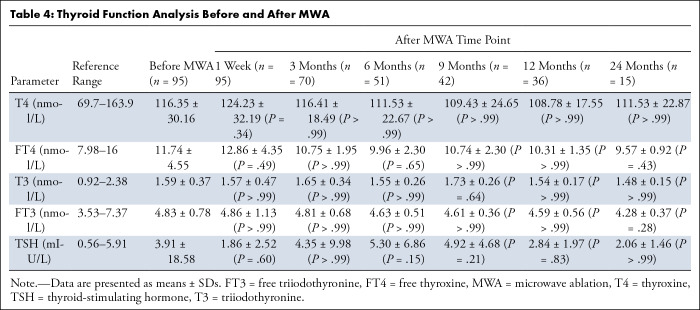
Thyroid Function Analysis Before and After MWA

### Complications

Of the 95 patients who underwent MWA, 93 had no postoperative complications. Two patients experienced mild neck swelling after MWA, which was alleviated with ice packs. During follow-up, none of the patients showed signs of recurrence or progression.

## Discussion

To our knowledge, this is the first study to evaluate the safety and efficacy of US-guided MWA in the treatment of Bethesda III thyroid nodules with negative molecular testing results. To date, guidelines issued by domestic and foreign thyroid associations, including the American Thyroid Association and the Korean Thyroid Radiology Society, have fully supported the role of thermal ablation in the treatment of benign and malignant thyroid nodules, including thyroid microcarcinoma, and metastatic cervical lymph nodes (14,15,18). However, the effectiveness and safety of MWA in the treatment of Bethesda III thyroid lesions have not been elucidated in large cohort samples. In our retrospective study of 95 patients, we found 1-year follow-up with a mean VRR of 83.54% ± 15.53, and 2-year follow-up with a mean VRR of 90.88% ± 13.59. The observed volume reduction was similar to that of benign nodules, ranging from 50% to 90%. A recent article in Italy also reported on the efficacy of Bethesda type III thyroid nodules, which showed a 1-year mean VRR of 62.00% ± 15.70 and a 2-year mean VRR of 66.90% ± 4.00 (19). There was no evidence of a difference in nodule volume at 3 months (*P* = .38) and 6 months (*P* = .50) after ablation compared with the preablation volume. This may be related to the immune function status of patients.

Several large-scale studies have revealed the genetic landscape of thyroid cancer (mainly *PTC*) in the Chinese population (4,20,21). *BRAF* is the most commonly mutated gene, with a mutation prevalence of approximately 76.0%, followed by *RET* rearrangement (7.6%) and *RAS*-driven mutations (4.1%) (4). Tumors with more somatic mutations are associated with poorer clinical characteristics, including older age at diagnosis, less differentiated tumors, larger tumor size, lymph node metastasis, and distant metastasis. The eight-gene panel we used in this study covers the most commonly mutated genes. A negative genetic result indicates low-risk stratification of the nodule. The current study provides evidence for the safety and feasibility of MWA in treating these low-risk nodules based on negative molecular profile, supporting its role in nodule management. The eight-gene panel detection is a common method of thyroid cancer-related gene detection in Chinese hospitals, especially in higher level hospitals. The sensitivity and accuracy of the eight-gene panel detection for the diagnosis of unidentified thyroid nodule were 92.10% and 92.68%, and the specificity and positive predictive value were 100%. The number of genes tested is a choice of balances between economic cost and diagnostic value (22). Our study demonstrated that, in the eight-gene negative atypia of undetermined significance nodules, MWA achieved significant VRRs of 83.54% and 90.88% at 12 months and 24 months, respectively. The incidence of postoperative complications was only 2% (two of 95). It is worth noting that this study did not report long-term complications (>2 years). Regarding the regrowth of benign thyroid nodules after thermal ablation, many studies have reported tissue regeneration caused by insufficient ablation edge 2–3 years after thermal ablation (23). There was no incidence of nodule regeneration after MWA in this study, which may be due to the limited patient number and the short follow-up time.

Our study reports the efficacy and safety of MWA treatment in patients with Bethesda III nodules, but it had important limitations. First, this was a retrospective study involving patients with a 2-year follow-up period. A longitudinal follow-up study recruiting a larger cohort of patients in a multicenter setting and with a longer follow-up period should be designed in the future to more accurately assess long-term outcomes. Second, as opposed to surgical resection, ablation therapy lacks pathologic reports after the procedure. Therefore, the potential risk for overlooking malignant lesions cannot be completely excluded, which is a limitation of ablation therapy. In addition, the molecular testing used in this study included only eight frequently mutated genes that are highly associated with thyroid cancer, and the lack of data for other related genes is another drawback of this study. Therefore, thorough preoperative evaluation and long-term active monitoring are crucial to comprehensively assess the safety and efficacy of MWA in treating Bethesda III thyroid nodules.

In conclusion, our work suggests that for Bethesda III thyroid nodules with negative molecular testing results, MWA is a safe and effective treatment and seems to be an ideal alternative approach for active monitoring or surgical resection, especially for patients who are not suitable for or strongly oppose surgery. This approach can preserve the thyroid while minimizing the surgical risks. However, because of the lack of pathology reports after ablation, it is not possible to determine whether the nodule is benign or malignant. Studies with larger sample sizes and longer follow-up periods can further determine the safety and feasibility of MWA treatment for Bethesda III thyroid nodules, which may optimize our current management strategy for these patients.
